# Sox2 Transcriptionally Regulates Pqbp1, an Intellectual Disability-Microcephaly Causative Gene, in Neural Stem Progenitor Cells

**DOI:** 10.1371/journal.pone.0068627

**Published:** 2013-07-16

**Authors:** Chan Li, Hikaru Ito, Kyota Fujita, Hiroki Shiwaku, Yunlong Qi, Kazuhiko Tagawa, Takuya Tamura, Hitoshi Okazawa

**Affiliations:** 1 Department of Neuropathology, Medical Research Institute, Tokyo Medical and Dental University, Bunkyo-ku, Tokyo, Japan; 2 Center for Brain Integration Research, Tokyo Medical and Dental University, Bunkyo-ku, Tokyo, Japan; Duke University Medical Center, United States of America

## Abstract

PQBP1 is a nuclear-cytoplasmic shuttling protein that is engaged in RNA metabolism and transcription. In mouse embryonic brain, our previous *in situ* hybridization study revealed that *PQBP1* mRNA was dominantly expressed in the periventricular zone region where neural stem progenitor cells (NSPCs) are located. Because the expression patterns in NSPCs are related to the symptoms of intellectual disability and microcephaly in *PQBP1* gene-mutated patients, we investigated the transcriptional regulation of *PQBP1* by NSPC-specific transcription factors. We selected 132 genome sequences that matched the consensus sequence for the binding of Sox2 and POU transcription factors upstream and downstream of the mouse *PQBP1* gene. We then screened the binding affinity of these sequences to Sox2-Pax6 or Sox2-Brn2 with gel mobility shift assays and found 18 genome sequences that interacted with the NSPC-specific transcription factors. Some of these sequences had *cis*-regulatory activities in Luciferase assays and *in utero* electroporation into NSPCs. Furthermore we found decreased levels of expression of PQBP1 protein in NSPCs of heterozygous Sox2-knockout mice *in vivo* by immunohistochemistry and western blot analysis. Collectively, these results indicated that Sox2 regulated the transcription of *PQBP1* in NSPCs.

## Introduction

PQBP1 is a novel protein identified by yeast two-hybrid screening that binds to the polyglutamine (polyQ) tract sequence [1], [2]. PolyQ sequence is shared by multiple transcription-related factors, such as TFIID/TATA-binding protein, androgen receptor, glucocorticoid receptor, octamer-binding factors, and CREB-binding factors, and by proteins related to a group of triplet-repeat diseases, which are called polyQ diseases, such asataxin-1, ataxin-2, atrophin-1/DRPLA protein, and huntingtin. These proteins belonging to the two groups were easily determined by putting the polyQ sequence into a BLAST search (http://blast.ncbi.nlm.nih.gov/Blast.cgi?PAGE=Proteins).The binding characteristics of PQBP1 suggests involvement of PQBP1 in the common pathology of multiple polyQ diseases [3], [4].Although PQBP1 is a small protein of 265 amino acids, it possesses two protein-interaction motifs, the WW domain (WWD) and the C-terminal domain (CTD). PQBP1 interacts with RNA polymerase II (Pol II) or WBP11/NpwBP/SIPP through WWD [4]–[6] and with a U5 spliceosome factor U 5–15 kD through CTD [7], [8].

PQBP1 is a nuclear-dominant nuclear-cytoplasmic shuttling protein that is engaged in RNA metabolism and transcription. PQBP1 functionally connects transcription and splicing when it is located in the nucleus [1], [4], [7]–[10], but, in abnormal conditions, it shifts to the stress granule in the cytoplasm and probably regulates RNA metabolism [11]. Although the dynamics and functional roles seem to be essential for all types of cells, previous analyses have indicated an unequal distribution of *PQBP1* mRNA and protein in different tissues and different cell types [1], [12]. In embryonic mouse brain, our previous *in situ* hybridization study revealed that *PQBP1*mRNA is dominantly expressed in the periventricular zone region where neural stem progenitor cells (NSPCs)are located [12].

PQBP1 has been implicated in both neurodegenerative diseases and developmental disorders.PQBP1 has been shown to interact with the two polyQ disease proteins, ataxin-1 and huntingtin [3], [4]. A European Consortium Study has revealed that mutations of the*PQBP1* gene are linked to intellectual disabilities (ID) that are characterized by mental retardation, microcephaly, and short stature [13]–[17]. Because the expression pattern in NSPCs is critical for an understanding of ID and microcephaly in*PQBP1* gene-mutated patients and of stem cell-related pathology in neurodegeneration, we investigated the transcriptional regulation of PQBP1 by NSPC-specific transcription factors in this study.

Especially, we focused on the role of Sox2 in the transcriptional regulation of PQBP1 in NSPCs. Sox2 is one of the most critical transcription factors for NSPCs. The stemness or pluripotency of NSPCs is mainly attributed to Sox2, which is clearly demonstrated in studies of induced pluripotent stem (iPS) cells [18]. The difference between totipotent embryonic stem (ES)/iPS cells and NSPC depends on the expression of Oct-3/4, which is expressed in ES/embryonic carcinoma (EC)/iPS cells [18]–[22], but it is rapidly downregulated during their differentiation by retinoic acid-repressive enhancer [19]. Actually, iPS cells were generated from NSPCs only with Oct-3/4 [23]. Sox2 forms a heterodimer with the POU transcription factors, including Oct-3/4 [24]–[26]. In this regard, the change in the Sox2 partner from Oct-3/4 to the NSPC-specific POU factor Brn2 or to another NSPC-specific transcription factor, such as Pax6, may switch on the expression of PQBP1 in NSPCs.

Sox2, which is a HMG-box-containing transcription factor that binds to the minor groove of DNA, forms a heterodimer with a POU transcription factor [27]. POU transcription factors are a group of AT-rich major groove-binding transcription factors, and they include Pit1, Oct-1, Oct-2, and Unc-86. In addition, the group includes Oct-3/4, which is a key transcription factor for ES cell differentiation [28] and iPS cell generation [18], and the other transcription factors.

Pax6 is expressed in apical and basal progenitor cells. Brn2 is highly expressed in NSPCs. Therefore, they could be the partner of Sox2 [27], [29]. Although Oct-3/4 has been shown to be expressed at high levels in stem cells ranging from ES cells to epiblasts in the neural lineage [19]–[22], it is downregulated to an extremely low level in NSPCs [30]. Furthermore, a conditional knockout of Oct-3/4 induced by nestin-Cre did not show morphological changes in the brain [31], and this was distinct from PQBP1-conditional knockout mice (our unpublished results). Thus, Oct-3/4 could be excluded as the candidate. Oct-6 might be a third candidate that is expressed in the glial cell lineage from NSPCs [32], [33] whereas the expression of PQBP1 is low in glial cells [1].

Hence, we employed the first 2 types of heterodimers (Pax6-Sox2, Brn2-Sox2), and screened for the genome sequence surrounding the *PQBP1* gene that were homologous to the Sox2-Pax6 or Sox2-Brn2-binding consensus sequence for their binding activity and transcriptional activity. Consequently we identified 18 genome sequences that interacted with the NSPC-specific transcription factors. *In utero* electroporation into NSPCs revealed that some of them had *cis*-regulatory activities *in vivo*. Furthermore, we found decreased expression of PQBP1 protein in NSPCs of heterozygous Sox2-knockout mice. All these results in this study supported that Sox2 regulates the transcription of *PQBP1* in NSPCs.

## Results

### PQBP1 Protein is Highly Expressed in NSPCs

**Figure 1:**
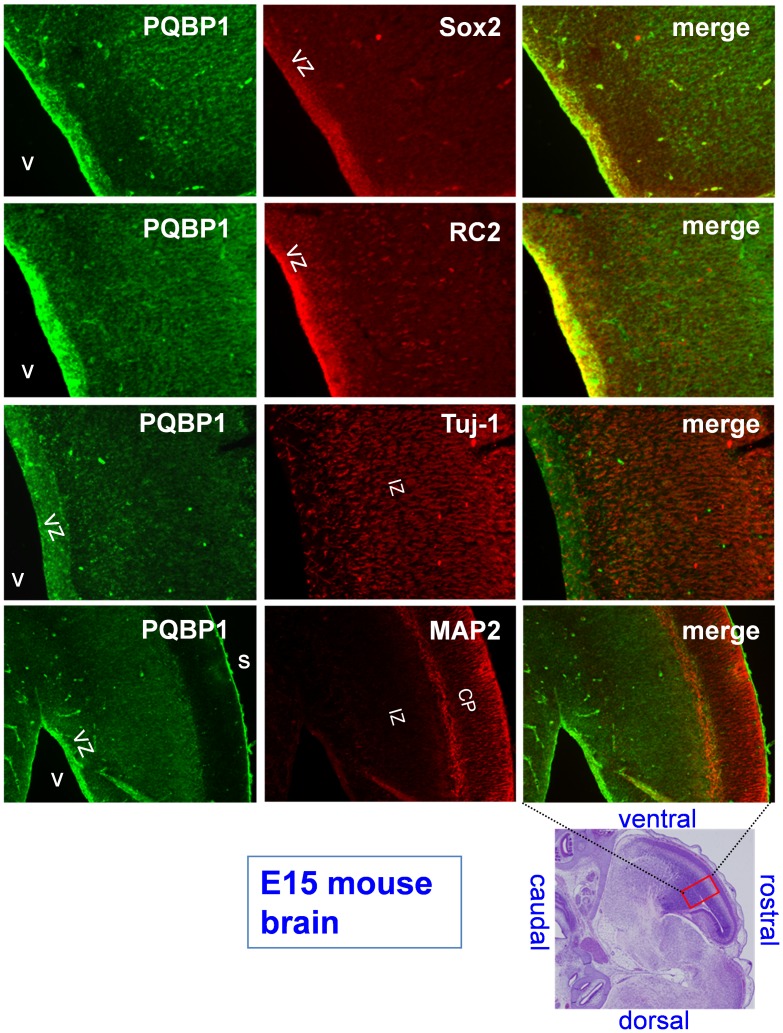
*PQBP1* is expressed in the ventricular zone. PQBP1 is expressed in the VZ of mouse embryonic brains at E15, which is the critical period for neuronal proliferation. Sagittal plane sections of the cerebral cortex were double-stained for PQBP1 and the following markers: Sox-2, for neural stem cells; RC2, specific for radial glia; Tuj-1, specific for differentiating immature neurons, and MAP2, to stain mature neurons. V, lateral ventricle; VZ, ventricular zone; S, cortical surface; IZ, intermediate zone; CP, cortical plate. Scale bar, 100 µm. The lower right panel shows the position of the magnified fields in the whole brain. Confocal microscopy (LSM510META, Carl Zeiss AG) with 10X lens was used to visualize the fluorescence.

We have previously shown with *in situ* hybridization that mouse *PQBP1* mRNA is expressed in mature neurons, but not in glia, in adult brains [1] and that *PQBP1* is dominantly expressed in the periventricular zone in embryonic brain [12]. The levels of expression were clearly higher in embryonic brain than in adult brain [12]. At the beginning of this study, we reexamined the previous findings at the protein level by immunohistochemisty. In E15 embryonic brain, *PQBP1*was highly expressed in NSPCs, but it was also weakly expressed in differentiating neurons (Figure 1). This finding reconfirmed the high levels of expression of PQBP1 in NSPCs that we found in our previous results.

**Figure 2:**
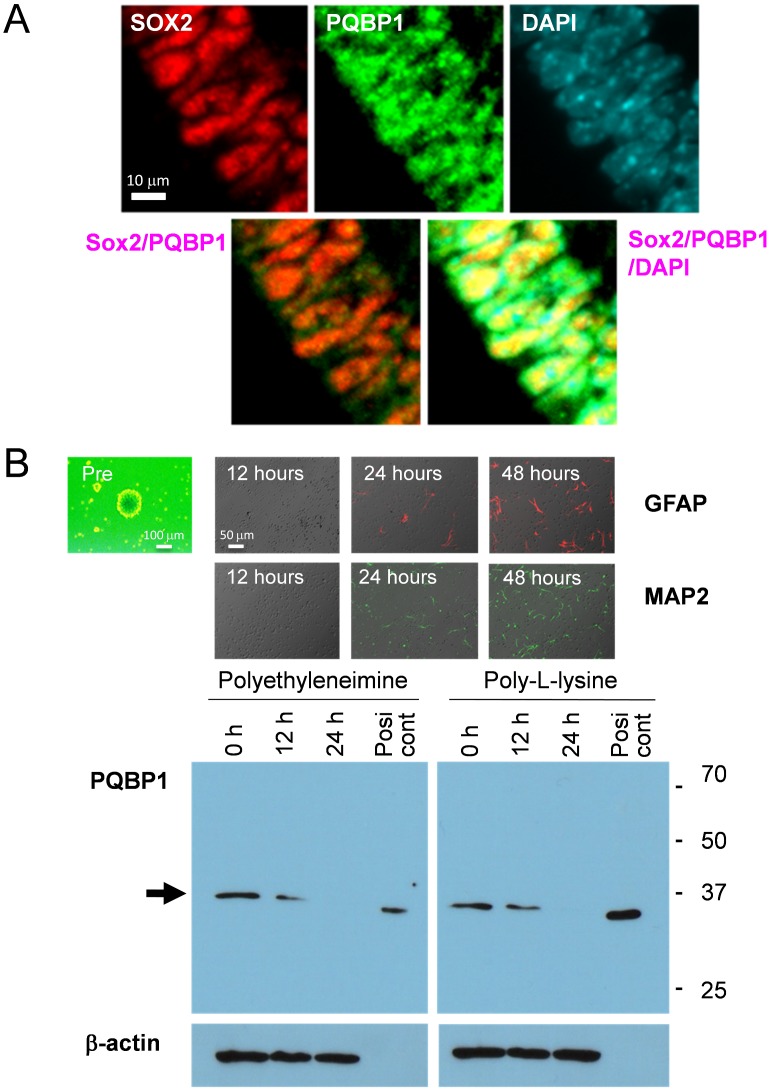
*PQBP1* is expressed in neural stem progenitor cells (NSPC). (A) Confocal microscopic analysis of VZ at E15 confirmed the colocalization of PQBP1 and Sox2 in the nuclei of NSPCs. Confocal microscopy (LSM510META, Carl Zeiss AG) with 40X water emersion lens was used to visualize the fluorescence. (B) NSPCs from E15 mouse were differentiated by plating them on dishes coated with polyethyleneimine or poly-L-lysine. The upper panels show the chronological expression of the differentiation markers. The lower panels show the downregulation of PQBP1 protein levels by the differentiation of NSPCs. The positive control is Drosophila Schneider cells that express PQBP1 and that do not show a band that is reactive to anti mammal beta-actin antibody. Digital images were captured by an Olympus IX71 microscope.

Although the PQBP1 protein has been shown to shuttle between the nucleus and the cytoplasm [1] and to move to a specific cytoplasmic domain when cells form stress granules under stressful conditions [11], PQBP1 concentrates in the nucleus due to its nuclear localization signal sequence in normal conditions [1]. An immunohistochemical analysis with confocal microscopy again demonstrated the *in vivo* expression of PQBP1 in periventricular NSPCs of E15 mouse embryo (Figure 2A).Moreover, PQBP1 was clearly colocalized with Sox2, which is a nuclear marker of NSPCs (Figure 2A). Immunohistochemistry with an anti-PQBP1 antibody showed PQBP1 protein expression in embryonic brain (Figure 1), and this protein expression was downregulated during the differentiation of NSPCs in a western blot analysis that was performed with primary NSPCs before and after differentiation by culture on polyethyleneimine and poly-L-lysine (Figure 2B).

### The *PQBP1* Gene is Surrounded by Multiple Sox2-Binding Sites

**Figure 3:**
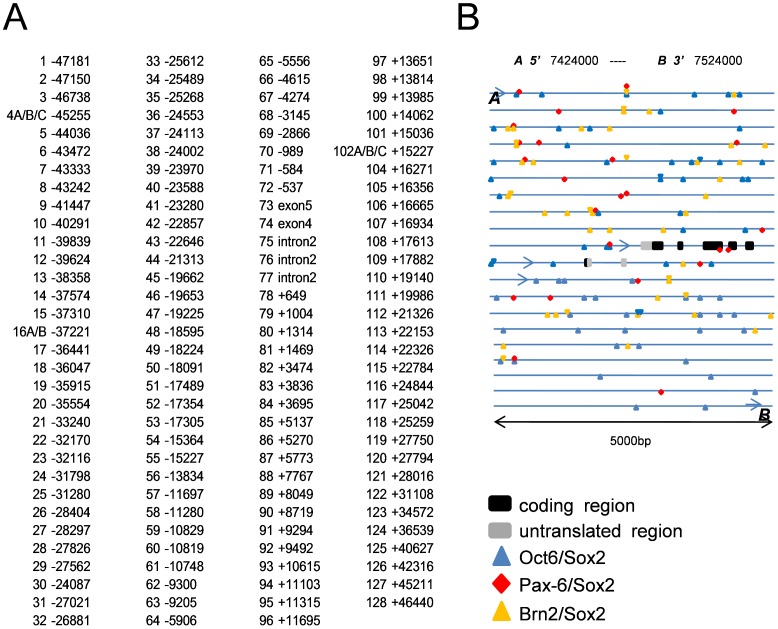
Possible *cis*-elements upstream and downstream of the *PQBP1* gene. (A) Possible *cis*-elements were selected by their similarity to the consensus binding sequence of Sox2-Brn2 or Sox2-Pax6. The positions on the genome of the first nucleotide in candidate *cis*-element sequences are shown. The distance from the 5′-end nucleotide of the *PQBP1* gene or from the 3′-end nucleotide of the *PQBP1* gene are shown as – or +, respectively. (B) The schematic shows the sites of the candidate *cis*-elements in the genomic region surrounding the *PQBP1* gene.

As introduced, we employed the Pax6-Sox2 and Brn2-Sox2 heterodimers and screened for the genome sequence surrounding the *PQBP1* gene. In the 57424000 to the 37524000 chromosomal region surrounding the *PQBP1* gene, we found 132 sequences that were homologous to the Sox2-Pax6 or Sox2-Brn2-binding consensus sequence (Figure 3 and Table 1). The criteria of consensus sequences are listed in Table 2. In addition, we found many binding consensus sequences for Sox2-Oct6, which will be discussed later.

**Table 1:** Probes for gel mobility shift assay.

number	position	sequence	number2	position2	sequence2
1	−47181	AAAATAACCA*aaat*AAAAAGTGT*ACAAT*TCAGTGGC	64	−5906	ATATATGC***ACAAA***CACTC**attt**ACATAAAATAA
2	−47150	GCCATAGA*ATTGT*ACTTCTGTCACCAGTTTTATTTTTTTATTT	65	−5556	GTACTTGGTGA**taat**TTC***ATTGT***CCACATGC
3	−46738	ACCTATAC*TTTGT*TTT*tatt*GCCTAGTAAT	66	−4615	GGAACTCCCTT**aaat**TTGG***TTTGT***TTATGTGT
4A	−45255	GACTTAATTGTGAGTCTCTGTAAAATTA*ACAAA*CAAGTTAC	67	−4274	GCATATGC***ATTGT***CTGTG***tatt***CTAAATGTAT
4B	−45255	AGTCTCTGTAaaatTA*ACAAA*CAAGTTAC	68	−3145	TTTGTTTG***TTTGT***TTAAAGG***attt***ATTTATTTAT
4C	−45255	TAAAATTA*ACAAA*CAAGTTAC*atta*TCTAATATACA	69	−2866	ACAAGTGA***TTTGT***CCTTA**TCAC**ATAGCTAGGAAAAGGGAAT
5	−44036	TGTAGGTC*ATTGT*GGCCTCAA*attt*GGTAATGTAA	70	−989	GAGATTTG***TTTGT***GCTATC***atta***GATCAATGAT
6	−43472	AGGACTTA*ATTGT*TCGC*attt*CTGTATTCTG	71	−584	GAGATTTG***TTTGT***GCTATC***atta***GATCAATGAT
7	−43333	CAACCACCTCTaaatGCCT*ACAAATTGT*ATGGTCAT	72	−537	GTTTGTTTTTTAAGGAACTG***taat***GA***ACAAA***TGTCAGGA
8	−43242	GCTGTAACAG*aata*CCTG*ACAAA*AGCAACTT	73	exon5	CTTGTCCTCAGCATCTATG**GTAA**CAC***ACAAA***TCCTGAAG
9	−41447	TCCTGCCACTTGGGTTGCAGTGAGG*ACAAT*GAAATGAG	74	exon4	GACTTTCTTGGCGGATTTG**GTAA**CG***ACAAA***GTTAGGAT
10	−40291	ACACACAC*ACAAA*GatttatttATTCATTTAAT	75	intron2	GCTCTGGGTA***taat***TA***ACAAT***AAAATGCA
11	−39839	GTGGGGTACAAaaatGGCCC*TTTGT*GATCAACT	76	intron2	CAGGTATG***ATTGT***CCA***tatt***GCTAATAAAC
12	−39624	AGTATGAG*TTTGT*ATATGTTG*atta*ACCAAACACT	77	intron2	TATGTCTA***TTTGT***TTTT***tatt***TTTTGAAGACT
13	−38358	TCTCCTGC*TTTGT*AGTCACAGAGTAGTCAAGAAGGGCT	78	+649	TCTTTTGA***TTTGT***CCG***attt***AGGTTTTAAA
14	−37574	GTCAGGAT***TTTGT***GTGTGG***atta***ACTGTATCAT	79	+1004	TGATAGTA***ACAAA***AACC**attt**TGTTTTTGGCT
15	−37310	AGATCTTT*ACAAA*TTTCTAT**atta**GAGTTTTTTGT	80	+1314	CATCCCAA***ATTGT***TTTC**TCAC**T***TTTGT***ACCACAGATAGAT
16A	−37221	GTGAACCCTTTCAGATTTAGTGACGTAGCTTC***ACAAA***GTGATTAA	81	+1496	AGAGTGCTGG***tatt***AAAAGGC***ACAAA***CTTTCTTT
16B	−37221	GTAGCTTC***ACAAA***GTGattaACACATGGATA	82	+3474	ACTATTTA***TTTGT***GTTTTCC***tatt***CACTTTACAT
17	−36441	TTTAAAGA***TTTGT***TTCTT***tatt***ATATGTAAGTA	83	+3836	GTCCAAGT***TTTGT***GACCCTTT***aata***CAGTTAGATC
18	−36047	CTTAGAATTAG**taat**TTCTTGAT***ATTGT***CTGTTAAG	84	+3965	TATGAATC***ACAAT***GTA***aata***TCTGTGTTTA
19	−35915	GAAGGAAA***ATTGT***TCCCTGAC***attt***TTGAACTGGG	85	+5137	ACATATGCA***ATTGT***TTCCTG*tatt*ATAGTAGAGAT
20	−35554	CTCAGAGCTAG**aaat**A***TTTGT***GGAATGGG	86	+5270	AGTTTAG***TTTGT***AGAGTAC**TTAC**CTAGCAGCACAAAGCCCTG
21	−33240	CAGAATCA***TTTGT***GTTTTCCC***attt***TTGCCAGGGG	87	+5773	TTATGACTGTT**taataat**ATAT***ATTGT***GTTCTGGC
22	−32170	TACATACAGCA**aaataat**C***TTTGT***AGGGGGGA	88	+7767	TGTTTTGT***TTTGT***TTTTTG***tatt***GGGAAGTGGG
23	−32116	AATCTCTATCTTACTTCTT**GTAA**GTGTGG***ACAAA***TGCAGGTT	89	+8049	CTACCACACCCAACCTCTA**GTAA**AGGTTT***ACAAA***ATGAATGA
24	−31798	CTTGTATC***TTTGT***ATGT**TCAC**AGAAAGCAAAGTAGCGTGG	90	+8719	TGCTGTTT***TTTGT***TACTG**TCAC**TAATTACATTTTAAATTAT
25	−31280	CAGATAAC***TTTGT***CCTTTTTG***attt***TGAGTGAAGA	91	+9294	*TTTGTTTGTTTGT*TTTTAAA***aata***AGGTCTCGGG
26	−28404	GTGGAGGC***TTTGT***GTAATCAGTCTGTGTA	92	+9492	ATATAATAAT***aaat***CTTTAAA***ACAAA***AAAAAAAA
27	−28297	CACACCTG***TTTGT***TTTCCACT**TCAC**AGAGGAGGCATAGGAAACT	93	+10615	ATAGAGGC***ACAAA***A***attt***CCAGAGACTGG
28	−27826	AAAAAAGATTC**taat**CTTT***TTTGT***GGTGGTGG	94	+11103	ATCTGTAAAGG**aaat**TAC***TTTGT***AGGA***aatt***TGATGGAACTG
29	−27562	AAAAACTT***TTTGT***TATTTC***attt***TTATTAGTGG	95	+11315	GGTTTTCT***TTTGT***ACACTT***aata***TCCGCTGGGG
30	−27087	TGTCTTCTCCC**taat**G***ATTGT***TGTATGAC	96	+11695	ACATAGACAC***aaat***AGCAG***ACAAT***GACCTTGA
31	−27021	TCCTCTCC***TTTGTTTTGT***CC**TCAC**ACCCCCAGCTGACCAGGTG	97	+13651	TAGATGCT***ACAAA***AGATACTG**atta**AAACCCAAATG
32	−26881	CGTTGCAC***ACAAA***CCTTA**attt**TCCAATCTATT	98	+13814	TTATGAAT***ACAAT***TC**atta**TAACCATGGAT
33	−25612	ATAGTACA***TTTGT***TGTC***atta***ACAAATCAGT	99	+13985	ATAT***AACAA***GA**taat**C***ATTGT***TTTG***aata***TTCCAAGGATTT
34	−25489	***TTGTT*** CCCTTTGACTTCTT**GTGA**CTTTA***ACAAA***TTCTAAAG	100	+14062	CAACCAAAAA***aaat***G***tatt***TT***ACAAA***AATCACAA
35	−25268	GTTTCTCTAATtaatAGACTTC***ATTGT***TCAAAACTatttTACATTTGTT	101	+15036	TGCCTCCT***TTTGT***GAGC*tatt*TATAATGCTAA
36	−24453	TGCATACA***TTTGT***AATTC***attt***AGGTGGTTTT	102A	+15227	GGATCCCCAC***aata***CAAAAAAA***ACAAA***AACAAAAC
37	−24113	TTTCATGG***TTTGT***TAGC***tatt***CTCTTATCAC	102B	+15227	AAAACAAA*ACAAA*ACTGG**attt**GGCGATTGTAT
38	−24002	AGGTTTTGATG**atta**TGA***ACAAA***GCTTCTGT	102C	+15227	ATTTGGCG***ATTGT***ATTAT***aatt***GTCTATCAGT
39	−23970	TTCAGGTT*TTTGT*AGGAACTTA***aatt***TTATGATTAG	104	+16271	CACAATTTTTA**taat**ATATA***ATTGT***TTCATCAA
40	−23588	TTTGTCAT***TTTGT***AGTGCTAGG***aatt***AAACCCATGT	105	+16356	ATTTTTTAAAA***aatt***AGACTACC***ACAAA***TCAACTGT
41	−23280	TTTGGAAA*ACAAA*AC**atta**ACCAAAATTTT	106	+16665	GCTTCCCTAG***aatt***GGAGAT***ACAAA***GGGCTCTG
42	−22875	GCTTCAGC***ATTGT***GA***aatt***GAATCATTGC	107	+16934	ACTAGTTTTC***tatt***ACT***ACAAA***TCTCAACT
43	−22646	AGGAGTGC***TTTGT***AAA***aata***ACTTCTATGT	108	+17613	CATCTTATCGT**taat**ATATACG***TTTGT***TACATATA
44	−21313	AAACCATGGGGGGTGGGCT**GTAA**AC***ACAAA***AAGGATTC	109	+17782	CTTTTTTT***TTTGT***ATGTG***attt***GAGGCAGAGT
45	−19662	AAATGTGCTT***tatt***TC***ACAAA***ATCAACAA	110	+19140	TTTATTTA***TTTGT***TT***tatt***TC***tatt***TTTCTTGGTGA
46	−19653	TTATTTCACA***aaat***CA***ACAAA***TCAAAAAG	111	+19986	TTCAACCT***TTTGT***TGTATC***attt***AAAAAACATT
47	−19225	CCACCATCTG***taat***GTCCTTGGC***ACAAT***AAAACCAA	112	+21326	TGTGTTCA***TTTGT***TTT***tatt***TTAGAAGGAGT
48	−18595	ATGAAATGGCT**aaat**CCTTTT***TTTGT***TGTTTGTT	113	+22153	ACTAAGTC***ATTGT***TT***attt***CCGTGCTGTT
49	−18224	CTCTCTGA***TTTGT***GAG***tatt***GACAGAGAAT	114	+22326	TGATTTTTGCA**aaat**TTTGT***TTTGT***TTCTTGAG
50	−18091	TTTGTAAG***TTTGT***ACC***attt***GGCCTCAGCC	115	+22784	CTGGCCAG***ACAAT***GACGAT**attt**TTTTCCTTCAC
51	−17489	GTCCGGCT***TTTGT***TG***attt***GCTTATCTAT	116	+24844	AAGGCCAGAC***taat***CT***ACAAA***GTGAGTTC
52	−17354	GGGGCAGC***ACAAA***T**attt**CTGTGAATGTA	117	+25042	GAAAA***ACAAA***G**attt**AGTATAAA
53	−17305	TGCTTGTG***ACAAA***TCTGTCA**attt**CTGGTGGGGGC	118	+25259	GACAAGGGTC***aatt***TGCTAG***ACAAA***GATCTTCA
54	−15364	AGGTGTTAAGTGTGGAGTC**GTGA**GAACA***ACAAT***CCAGATAG	119	+27750	CCGATCGC***TTTGT***TCTAC***attt***TATGTTCTAT
55	−15227	GTAGATTA***TTTGT***CCACCAT**TCAC**AAGCACTGAGTTCAATAAC	120	+27794	TCCACACTCTA**taat**CTCAC***ATTGT***TTGGCACC
56	−13834	ACACTTAT***ACAAT***AATAGT**attt**ATTAATAGTAT	121	+28016	GTGCTCAA***TTTGT***GTTAGC***ta*** **TTAC**TAGCAAGTCATTAACTCAC
57	−11697	CCTAAGATTCT**taat**CTGAC***ATTGT***AGTCCCTA	122	+31108	GAAAAGGA***TTTGT***ACTTA***aata***AGCACCTGGC
58	−11280	TGAGATGGAGG**aaat**AAGTCTT***ATTGT***TGTTCTCC	123	+34572	AGTGCTAT***TTTGT***CGAGA***aatt***GTAGAAGACA
59	−10829	AGCTTTTA***ACAAT***TTA**attt**TGTCTCTACCT	124	+36539	CTTATGCTTC***tatt***CTAC***ACAAT***TCCACAAC
60	−10819	AATTTAAT***TTTGT***CTCTACCTT**TCAC**GCTTCTCCTTTGCATTTTC	125	+40627	GGGATGCA***ATTGT***CAG**TCAC**CTTTCTTGAGCCTGTGGGA
61	−10748	TAAATTTTTC***taat***CCTG***ACAAA***TCTCTTCA	126	+42316	AATGTTGTCA***taat***TGAAAAAG***ACAAA***GCACTAAT
62	−9300	TTTAA***ACAATTGT***CCCATTC***atta***TATTGCAACA	127	+45211	TTTTGTTT***TTTGT***TTAAAGC*aata*ACATATTCAG
63	−9205	AGAAATCCTC***aatt***TCCTTATCC***ACAAA***ATGGAC**attt**TCCTAGTACCT	128	+46440	AATATCAGTGTAATG***atta***ACAG***ACAAT***GGCAGAGG

The sequences of oligonucleotide probes used for gel mobility assay are listed. Consensus sequences of transcriptional factors are indicated with bold letters. Each consensus sequences are presented in table 2. Sox2 (capital, italic), Brn2 (miniscule), Pax6 (capital), Oct6 (miniscule, italic). The number and position correspond to the information in Fig 3.

**Table 2:** Consensus sequence and spacing.

protein	consensus binding motif	heterodimer	spacing(N)
Sox2	***ATTGT, TTTGT, ACAAT, ACAAA***		
Brn2	**aaat, taat, attt, atta**	Sox2/Brn2	1≤N≤11
Pax6	**TCAC, TTAC, GTGA, GTAA**	Sox2/Pax6	2≤N≤9
0ct6	***aata, aatt, attt, atta, tatt, aatt, aaat, taat, aata***	Sox2/Oct6	2≤N≤10

The consensus sequence and spacing are listed for each transcriptional factor. “Capital”, “miniscule”, “italic” corresponded to the information in Table 1.

**Figure 4:**
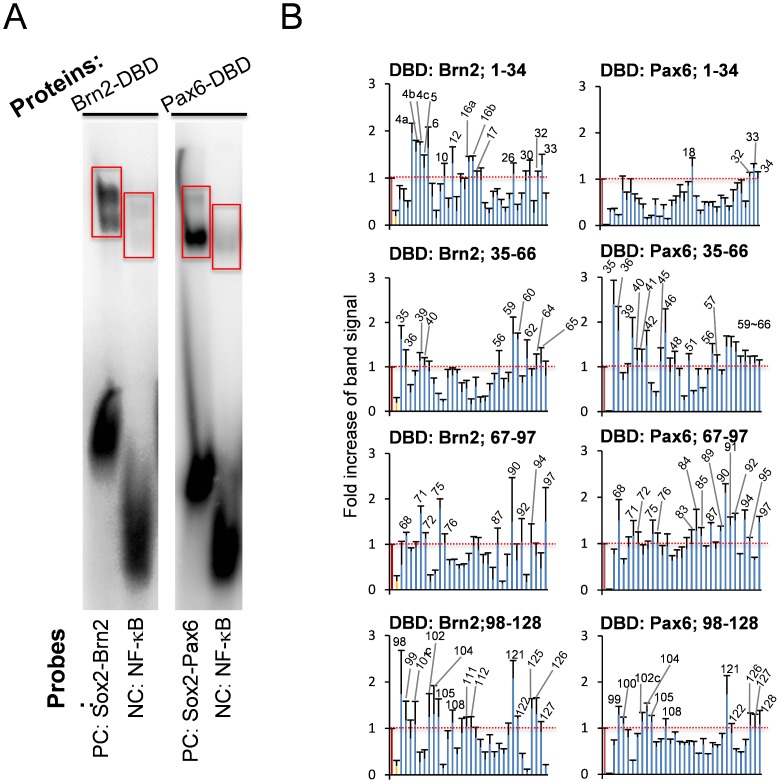
Screening of *cis-*elements by a gel mobility shift assay with the Brn2 or Pax6 DNA-binding domain (DBD). The left panel shows a representative gel mobility shift of the Sox2-Brn2 or Sox2-Pax6 consensus probe by Brn2-DBD or Pax6-DBD. A NF-κB consensus probe was used as a negative control. The probe sequences were the following: Sox2-Brn2: GGGTAGTGTGGACAAAAGGCAATAATTAGCATGAGAATC, Sox2-Pax6: GGGAAATATTCATTGTTGTTGCTCACCTACCATGGA, and NF-κB: GGGAGTTGAGGGGACTTTCCCAGGC. The right graphs show the radioactivity in the expected area of the gel shift of Brn2-DBD or Pax6-DBD (surrounded by red line). The values indicate the fold increase of the band intensity when the intensity of the positive control was set as 1.0.

**Figure 5:**
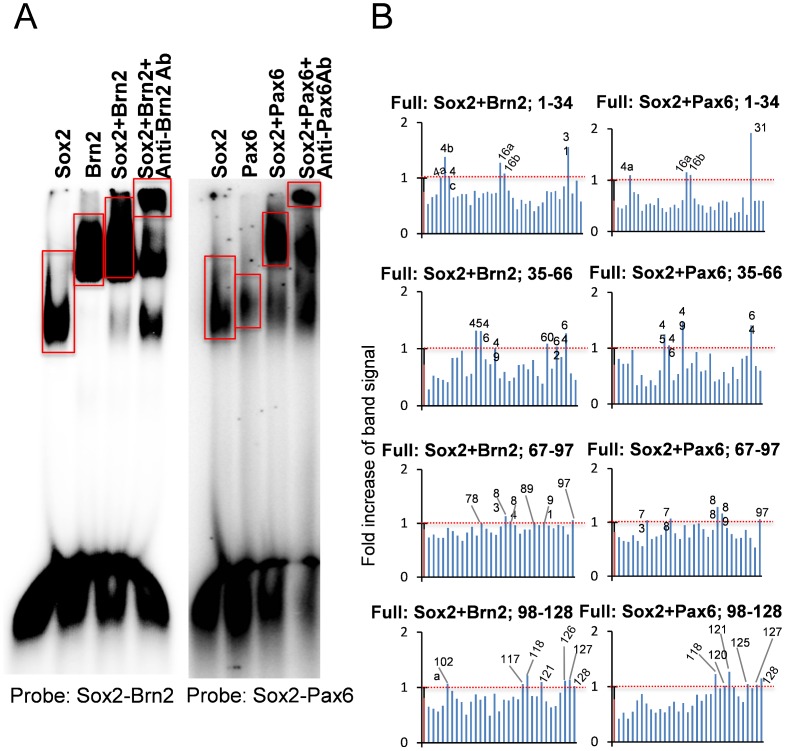
Screening of *cis*-elements by a gel mobility shift assay with Sox2-Brn2 or Sox2-Pax6 full-length protein heterodimer. The left panel shows a representative gel mobility shift of Sox2-Brn2 or Sox2-Pax6 consensus probes by the heterodimer of Sox2-Brn2 or Sox2-Pax6 full-length proteins. A supershift of the band with anti-Brn2 or -Pax6 antibody is also shown in the 3^rd^ lane. The probe sequences were the following: Sox2-Brn2: GGGTAGTGTGGACAAAAGGCAATAATTAGCATGAGAATC andSox2-Pax6: GGGAAATATTCATTGTTGTTGCTCACCTACCATGGA. The right graphs show the radioactivity in the expected area of the gel shift of Sox2-Brn2 or Sox2-Pax6 full-length proteins. The threshold is the average signal intensity plus 1× standard deviation.

From these sequences, we synthesized oligonucleotide probes and performed gel mobility shift assays. First, we checked the soundness of our method with positive controls of the *cis*-element (Figure 4) and with GST-only proteins (Supplementary Figure 1). We next screened the 132 probes with GST-fusion proteins of the Brn2 or Pax6 DNA-binding domain and found68positive probes (Figure 4 and Supplementary Figure 2B). The determination of positive probes was based on the radioactivity of the expected position of the band and on their statistical differences compared to the negative controls (Figure 4). These were termed first-positive probes. Similarly, with the gel shift assay, we screened the candidate probes by the heterodimers of the full-length Sox2-Brn2 or Sox2-Pax6 proteins (Figure 5 and Supplementary Figure 2A). The second screening selected 28 second-positive probes (Figure 5). Through the comparison of first-positive and second-positive probes (Table 3), we selected double-positive probes, the binding of which to the Sox2 heterodimer was considered reliable. Consequently, we selected 18 probes (Table 4). Double-positive probes for Sox2-Pax6 were also positive for Sox2-Brn2, while double positive probes for Sox2-Brn2 were not always positive for Sox2-Pax6 (Table 3). Since both Brn2 and Pax6 prefer AT-rich sequences and Sox2 consensus sequences in minor groves are not extremely specific, such overlapped binding of the two heterodimers might have occurred.

**Table 3:** Results of gel mobility shift assay.

probe No.	BD-Brn2	Sox2/Brn2	BD-Pax6	Sox2/Pax6	BD-Oct6	probe No.	BD-Brn2	Sox2/Brn2	BD-Pax6	Sox2/Pax6	BD-Oct6
1	−	−	−	−	−	64	+	+	+	+	+
2	−	−	−	−	−	65	+	−	+	−	+
3	−	−	−	−	−	66	−	−	+	−	+
**4a**	**+**	**+**	−	**+**	**+**	67	−	−	−	−	−
**4b**	**+**	**+**	−	−	**+**	68	+	−	+	−	+
**4c**	**+**	**+**	−	−	**+**	69	−	−	−	−	+
5	+	−	−	−	−	70	−	−	−	−	+
6	+	−	−	−	+	71	+	−	+	−	+
7	−	−	−	−	−	72	+	−	+	−	+
8	−	−	−	−	−	73	−	−	−	−	−
9	−	−	−	−	−	74	−	−	−	−	−
10	+	−	−	−	−	75	+	−	+	−	+
11	−	−	−	−	−	76	+	−	+	−	+
12	+	−	−	−	−	77	−	−	−	−	−
13	−	−	−	−	−	78	−	+	−	+	+
14	−	−	−	−	−	79	−	−	−	−	−
15	−	−	−	−	−	80	−	−	−	−	+
**16a**	**+**	**+**	−	**+**	**+**	81	−	−	−	−	−
**16b**	**+**	**+**	−	**+**	**+**	82	−	−	−	−	+
17	+	−	−	−	+	**83**	−	**+**	**+**	**+**	**+**
18	−	−	+	−	+	**84**	−	**+**	**+**	**+**	−
19	−	−	−	−	−	85	−	−	+	−	−
20	−	−	−	−	−	86	−	−	−	−	−
21	−	−	−	−	−	87	+	−	+	−	+
22	−	−	−	−	−	88	−	−	−	+	−
23	−	−	−	−	−	**89**	−	**+**	**+**	**+**	−
24	−	−	−	−	−	90	+	−	+	−	+
25	−	−	−	−	−	91	−	+	+	−	+
26	+	−	−	−	+	92	+	−	+	−	+
27	−	−	−	−	−	93	−	−	−	−	−
28	−	−	−	−	−	94	+	−	+	−	+
29	−	−	−	−	+	95	−	−	+	−	+
30	+	−	−	−	+	96	−	−	−	−	−
31	−	+	−	+	−	**97**	**+**	**+**	**+**	**+**	**+**
32	+	−	+	−	+	98	+	−	−	−	+
33	+	−	+	−	+	99	+	−	+	−	+
34	−	−	+	−	−	100	−	−	+	−	+
35	+	−	+	−	+	101	+	−	−	−	+
36	+	−	+	−	+	102a	−	+	−	−	−
37	−	−	−	−	−	102b	−	−	−	−	−
38	−	−	−	−	+	102c	+	−	+	−	+
39	+	−	+	−	+	104	+	−	+	−	+
40	+	−	+	−	+	105	+	−	+	−	+
41	-	−	+	−	+	106	−	−	−	−	−
42	−	−	+	−	−	107	−	−	−	−	−
43	−	−	−	−	−	108	+	−	+	−	+
44	−	−	−	−	−	109	−	−	−	−	−
**45**	−	**+**	**+**	**+**	**+**	110	−	−	−	−	+
**46**	−	**+**	**+**	**+**	**+**	111	+	−	−	−	+
47	−	−	−	−	+	112	+	−	−	−	+
48	−	−	+	−	−	113	−	−	−	−	−
49	−	+	−	+	−	114	−	−	−	−	−
50	−	−	−	−	−	115	−	−	−	−	−
51	−	−	+	−	−	116	−	−	−	−	−
52	−	−	−	-	−	117	−	+	−	−	−
53	−	−	−	−	−	118	−	+	−	+	−
54	−	−	−	−	+	119	−	−	−	−	−
55	−	−	−	−	+	120	−	−	−	+	+
56	+	−	+	−	+	**121**	**+**	**+**	**+**	**+**	**+**
57	−	−	+	−	+	122	+	−	−	+	+
58	−	−	−	−	+	123	−	−	−	−	−
59	+	−	+	−	+	124	−	−	−	−	−
**60**	**+**	**+**	**+**	−	**+**	125	+	−	−	+	+
61	−	−	+	−	+	**126**	**+**	**+**	**+**	**+**	**+**
**62**	**+**	**+**	**+**	−	**+**	**127**	**+**	**+**	**+**	**+**	**+**
63	−	−	+	−	+	**128**	−	**+**	**+**	**+**	−

The results in the gel mobility shift assay are summarized. The positive result is indicated with “+”. The negative result is indicated with “-“. The bold characters indicate “double positive” probe with which both DNA-binding domain (BD-Brn2 or BD-Pax6) and Sox2 heterodimers (Sox2/Brn2 or Sox2/Pax6) interact. The “probe No.” corresponds to Fig. 3.

**Table 4:** The list of “double positive” probes.

double positive probes selected for Luc assay : (Bold in Table 3)					
4a	4b	4c	16a	16b	45
46	60	62	64	83	84
89	97	121	126	127	128

**Figure 6:**
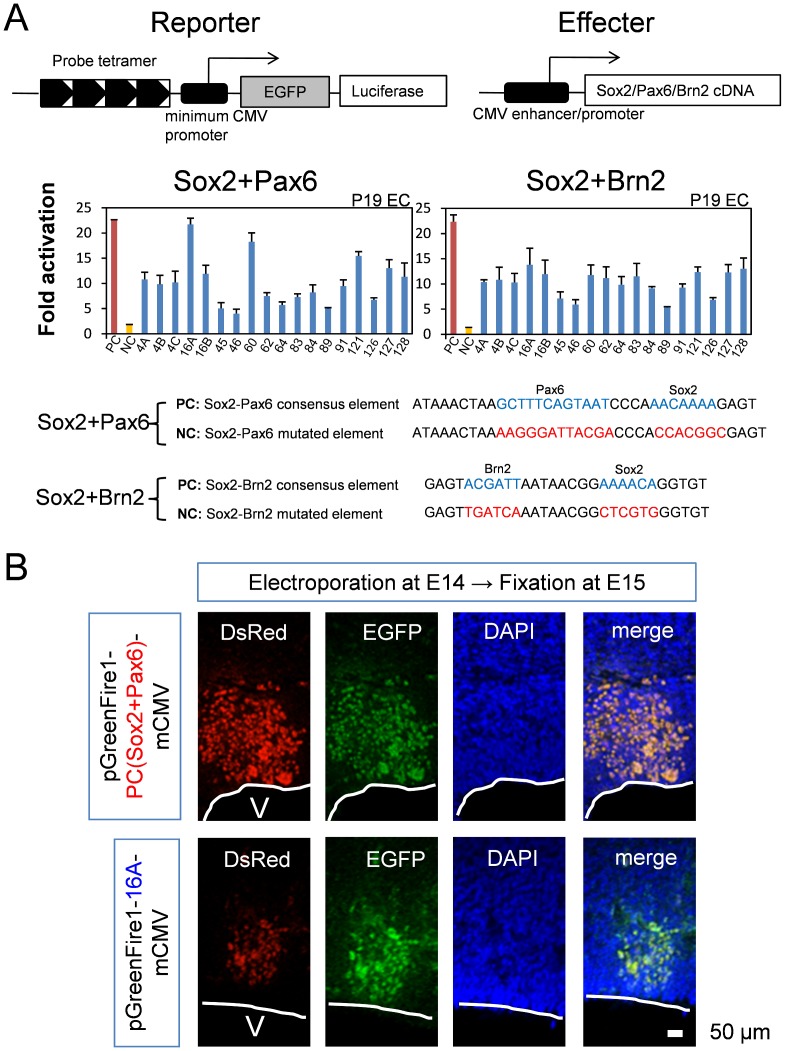
Screening of *cis-*elements by luciferase assay. (A) The double-positive *cis*-elements (Table 1) were subcloned into a reporter plasmid (pGreenFire1-mCMV), and their transcriptional activity was tested by cotransfection with effecter plasmids (Sox2+Pax6 or Sox2+Brn2) into P19 cells. Fold activation was calculated in comparison to the activity of the negative control reporter plasmid possessing a nonsense sequence as the *cis*-element. We simultaneously tested positive controls (PC) and negative controls (NC) that possess Sox2-Pax6 or Sox2-Brn2 consensus and mutated elements, respectively. Red and yellow bars represent transcriptional activity of PC and NC, respectively. (B) The transcriptional activity of the *cis*-element (P16A) in neural stem/progenitor cells *in vivo* was examined by *in utero* electroporation of the reporter plasmid. Electroporation was performed on E14 and sampling was done on E15. The positive control reporter plasmid, pGreenFire1-PC (Sox2+Pax6)-mCMV, possesses a Sox2-Pax6 consensus sequence. pGreenFire1-16A-mCMV was generated by inserting the 16A oligpnucleotides:GTGAACCCTTTCAGATTTAGTGACGTAGCTTCACAAAGTGATTAA into pGreenFire1-mCMV. Confocal microscopy (LSM510META, CarlZeiss AG) with 40X water emersion lens was used to visualize the fluorescence. Green fluorescent protein signals were detected in NSPCs, even though the DsRed signals were weaker than the positive control, indicating that 16A possessed strong enhancer activity *in vivo*.

The double positive probes in Table 3 are listed. They were employed for luciferase assay (Fig. 6).

In addition, we performed a gel shift assay with Sox2-Oct6 (Supplementary Figure 3). The results suggested that this complex could bind to part of the candidate *cis*-elements. However, Oct-6 expression in NSPCs has not been confirmed in previous studies, and our immunohistochemistry did not support Oct-6 expression in NSPCs (data not shown). In addition, as already mentioned we previously confirmed that PQBP1 is not highly expressed in glia compared to neurons [1], indicating that Sox2-Oct6 does not contribute to the transcriptional control of *PQBP1*. Thus, we did not use the complex in the following experiments.

In this study, we did not perform ChIP assay because the gel shift assays were multiplexed. However, ChIP assay might have strengthened our results, even though the bases of gel shift assay (supershift assay) and ChIP assay are overlapped.

### PQBP1 is Regulated by Sox2

The double-positive sequences were distributed both upstream and downstream of the *PQBP1* gene (Supplementary Figure 4). Four repeats of probe sequences in tandem were subcloned into the upstream sequence of the luciferase gene in reporter plasmids, and their *cis*-activities were tested in a luciferase assay. We cotransfected reporter and effector plasmids (Sox2+Pax6 or Sox2+Brn2) into P19 cells (Figure 6A). P19 cells, which are equivalent to epiblasts, were selected as Sox2 and PQBP1-expressing cells [1].

The luciferase assay showed obvious transactivation by the combination of transcription factors on the 18 *cis*-elements. The cotransfection of Sox2 and Pax6 showed remarkable transactivation with the #16A and #60 *cis*-elements (Figure 6A). The cotransfection of Sox2 and Brn2 showed less remarkable transactivation, and most *cis*-elements showed definite higher transactivation than that of negative controls (Figure 6A). In these experiments, we used positive controls (PC) and negative controls (NC) possessing Sox2-Pax6 or Sox2-Brn2 consensus and mutated elements, respectively (Figure 6A).

Next, we tested whether the *cis*-element that was selected by the luciferase assay could induce transactivationin NSPCs *in vivo* (Figure 6B). We performed electroporation of the reporter plasmids (pGreenFire1-PC-mCMV and pGreenFire1-16A-mCMV), which possessed enhanced green fluorescent protein (EGFP) gene cassettes downstream of the *cis*-element (Figure 6A), into the brain of E14 mouse embryos and tested whether the reporter EGFP gene was expressed. As a marker of transfected cells, we cotransfected pLVSIN-CAG-pur-DsRed.When we used a positive control reporter plasmid (pGreenFire1-PC-mCMV), we observed EGFP signals in transfected cells (Ds-Red-positive cells) (Figure 6B), indicating that the positive control *cis*-element for Sox2-Pax6 actually worked *in vivo*. Similarly, we observed green fluorescence in red cells when we transfected pGreenFire1-16A-mCMV (Figure 6B and Supplementary Figure 5A). EGFP signal was detected in some cells outside of ventricular and subventricular zones, probably because NSPCs were differentiated before EGFP protein was completely degraded. Notably, the ratio between GFP and DsRed was clearly higher with pGreenFire1-16A-mCMV than with pGreenFire1-PC-mCMV, indicating a strong enhancer activity of 16A sequence. Meanwhile, in untero electroporation of a negative control plasmid, pGreenFire1-NC (Sox2+Pax6)-mCMV did not produce EGFP signals, indicating that the result was not artifact (Supplementary Figure 5B). All the results supported that the *cis*-element we selected possessed enhancer activity in NSPCs *in vivo*.

### Sox2 Knockout Decreased PQBP1 *in vivo*

**Figure 7:**
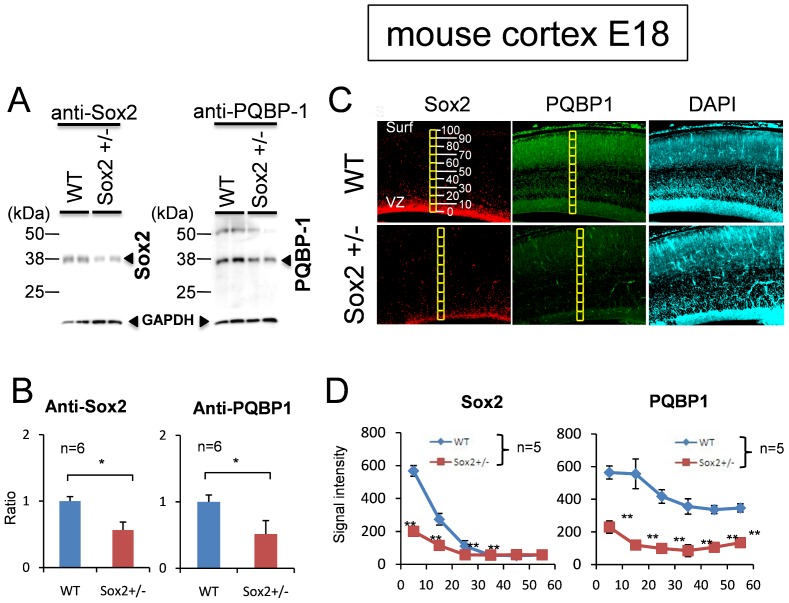
Sox2 regulates PQBP1 expression *in vivo*. (A) Western blot of primary-cultured neural stem progenitor cells from two Sox2+/− mice and two littermate mice (WT) (E14). Sox2 and PQBP1 proteins were both reduced. (B) Ratios of western blot signal intensities (Sox2/internal control and PQBP1/internal control) were quantified. Asterisk indicates statistical differences (N = 6, p<0.05 by Student’s *t*-test). (C) Immunohistochemistry of Sox2 and PQBP1 in Sox2+/− and littermate (WT) mice (E18). Digital images were captured by an Olympus IX71 microscope. (D) Quantification of the intensity of Sox2 and PQBP1 immunostain signals from ventricular surface to cortical surface. Cortex at E18 were divided into 10 square areas from ventricular surface (0%) to cortical surface (100%) as shown in Figure 7C. The total signals were acquired in each square, background signals (mean signals in ventricles, N = 5) were subtracted from them, and the mean and SD values were calculated (N = 5). Asterisks indicate statistical differences (p<0.01 by Student’s *t*-test).

Finally, we tested whether a decrease of Sox2 affected PQBP1 expression *in vivo*. We used heterozygous Sox2-knockout (Sox2+/−) mice in which the Sox2 expression was almost 50% of control mice (Figure 7A, B). The levels of expression of PQBP1 in Sox2+/− NSPCs were about 50% of the control, judging from the western blot analysis of the primary NSPCs prepared from E18embryonic brains (Figure 7A, B), and about 30% of the control, judging from the immunohistochemical staining of the embryonic brain (E18) (Figure 7C, D). These results strongly supported that Sox2 regulated *PQBP1* expression in NSPCs not only *in vitro,* but also *in vivo*.

## Discussion

In this study, we revealed the involvement of Sox2 in the transcriptional regulation of *PQBP1*in NSPCs. This conclusion was supported by multiple findings *in vitro* and *in vivo*. First, PQBP1 was coexpressed with Sox2 in NSPCs (Figure 1, 2). Second, Sox2 bound to multiple *cis*-elements that possessed transcriptional activities (Figure 3–6). Third, Sox2 haploinsufficiency reduced the levels of expression of PQBP1 *in vivo* (Figure 7). All of these findings indicated that PQBP1 was the downstream target of Sox2 in NSPCs.

Sox2 is a central player in various types of stem cells including ES cells. Therefore, one may ask why PQBP1 is not highly expressed in embryo before E11 [12] or in ES cells (our unpublished data). Actually Sox2 forms a heterodimer with Oct-3/4 in ES cells [34] and regulates toti-potency as shown in iPS cells [35]. However, the inner cellular mass that corresponds to ES cells does not need Sox2 for pluripotency, although it has been suggested that maternal Sox2 protein that remains in the cells compensates for the inactivation of Sox2 by gene targeting [36]. Considering that the expression of Oct-3/4 is extremely low in NSPCs [30], [37], that the expression of Sox2 is found in epiblasts and NSPCs, and that Oct-3/4 is sufficient for reprogramming from NSPCs [23], the demand for Sox2 might be larger in NSPCs.

Human ID diseases that are linked to *PQBP1*, such as Renpenning syndrome, Golabi-Ito-Hall syndrome, and Southerland-Haan syndrome, show multiple symptoms, such as microcephaly, short stature, lean body, and small testis [23]. The molecular pathways that result in each symptom are not yet understood. We have shown previously that the levels of expression of PQBP1 are correlated with the behavioral abnormalities. In *PQBP1-*knockdown mice in which the PQBP1 protein levels are reduced to 50% in the adult brain, we observed that anxiety-related cognition is impaired [38]. A consistent result was obtained in that Drosophila hypomorph mutants of PQBP1 homologs possessed a lower learning ability [39]. In addition, PQBP1 expression in NSPCs could be related to microcephaly, which is another phenotype of PQBP1-linked ID patients. With respect to these clinical aspects, the conclusion of this study that Sox2 controls the transcription of *PQBP1* gene in NSPCs is critical.

There are various approaches to possibly modify the symptoms of PQBP1-linked ID patients. Because there are various alternative splicing isoforms of PQBP1 and the disease mutations of PQBP1 are complex, some isoforms that partially retain the functions of the full-length PQBP1 might be expressed in patients. Therefore, in analogy with Duchenne muscular dystrophy, increasing the levels of expression of partially active isoforms may alleviate the symptoms of patients. It would be also possible to enhance PQBP1 expression at transcription level in order to increase functional PQBP1. In this regard, the findings of this study might be useful for the development of a novel therapeutic approach that can be used to modulate the levels of expression of PQBP1 through Sox2.

Our findings were also important for developmental biology. Sox2 is now recognized as a key transcription factor that is involved in multiple neurological diseases [40]. This in turn indicates that Sox2 plays multiple physiological roles during development and in adulthood for normal brain morphology and function. Our results indicated that*PQBP1* is a target molecule of Sox2, which is part of Sox2 developmental functions.

The homozygous deletion of Sox2 is mostly lethal in mice, but the phenotype that is observed with other types of Sox2 mutations are thought to partially be mediated by PQBP1. An important finding from the Sox2 conditional depletion in the brain is neurodegeneration [41]. Those authors made 2 types of Sox2-mutant mice. In one model involving β-geo knockin mice, they observed Sox2 expression in adult neurons and adult neurogenic regions and reduced cellular proliferation in the hippocampus and periventricular area [41]. They also observed in another mutated mouse model, in which a neural cell-specific enhancer was deleted, that Sox2 depletion in adult neurons resulted in the abnormal morphology of neurons and cytoplasmic inclusion body formation [41]. Considering these experimental results, *PQBP1* could be a target of Sox2 not only during development, but also in adulthood. The role of PQBP1 in adult neurodegeneration could be our next target of investigation. Insufficiency of the Sox2-PQBP1 axis could be a cause of degeneration, and its upregulation might be used as a therapy of degeneration in the future as a regenerative approach, as suggested previously [42].

## Methods

### Plasmid Construction

For construction of the GST-fusion vectors, cDNA fragments of full-length Sox2 (forward 5′-ATGTATAACATGATGGAGACGGAGC-3′, reverse 5′-TCACATGTGCGACAGGGGCAG-3′), full-length Brn2 (forward 5′-ATGGCGACCGCAGCGTCTAAC-3′, reverse 5′-TCACTGGACGGGCGTCTGCAC-3′), the DNA-binding domain of Brn2(forward 5′-GAGGCAGACTCATCCTCGGGCA-3′, reverse 5′-TGGCGTGTCCCTACTACCCCCATA-3′), full-length Pax6 (forward 5′-ATGCAGAACAGTCACAGCGG-3, reverse5′-TTACTGTAATCGAGGCCAGTACTGAGA-3′), the DNA-binding domain of Pax6 (forward 5′-ATCAGTTCTAACGGAGAAGAC-3′, reverse 5′-GCTGCTGATAGGAATGTGAC-3′), and the DNA-binding domain of Oct6 (forward 5′-GAGACCGACTCGTCCAGCGGCA-3′, reverse 5′-CCCCAGCTCCCCAGGCGCATAAA-3′) were amplified by polymerase chain reaction from B6 wild-type mouse embryonic brain RNA and subcloned into pGEX-6P-1 (GE Healthcare, Buckinghamshire, UK) between BamHI-EcoRI (for Sox2, Brn2, and Oct6 full-length and DNA-binding domains) or BamHI-XhoI (for Pax6 full-length and DNA-binding domain). The sequences were confirmed after subcloning.

*For Mammalian expression vectors*, full-length Sox2, Brn2, and Pax6 cDNAs cleaved out from pGEX-6P-1with BamHI and NotI were subcloned into pBS II SK+ (Agilent Technologies, Inc., Santa Clara, CA, USA) and again subcloned into pCI-neo (Promega Corporation, Madison, WI, USA) with single SalI site (Sox2 and Brn2) or SalI and NotI sites (Pax6).

### Expression and Purification of GST-fusion Proteins

GST-fusion proteins of full-length and the DNA-binding domain of transcription factors were induced in *Escherichia coli* cells (BL21) by 0.1 mM IPTG for 6 h at 25°C. The cells were collected, washed twice with PBS, resuspended in lysis buffer [10 mM Na_2_HPO_4_, 1.8 mM KH_2_PO_4_, 500 mM NaCl, 2.7 mM KCl,10% glycerol, 1 mM DTT, 0.5 mM PMSF, and protease inhibitor “complete” (GE Healthcare)], and sonicated. GST-fusion proteins were purified by glutathione-sepharose 4B according to the commercial protocol (GE Healthcare). Sodium dodecyl sulfate (SDS)-polyacrylamide gel electrophoresis was performed in order to confirm the sizes of the GST-fusion proteins. Western blot analysis was performed in order to test the validity of the proteins with anti-Sox2 (ab5603, EMD Millipore Corporation, Billerica, MA, USA), anti-pax6 (Developmental Studies Hybridoma Bank, Iowa City, Iowa, USA), anti-Brn2(sc-6029, Santa Cruz Biotechnology, Inc., Santa Cruz, CA, USA), and anti-GST (sc-459, Santa Cruz Biotechnology, Inc.) antibodies.

### Gel Mobility Shift Assay

Sense and antisense oligonucleotides at 5′ terminals containing 5 dGs at the 5′-end were annealed and radiolabeled with a Random Primer DNA Labeling Kit (Takara Bio Inc., Shiga, Japan) with [α-32P]dCTP (MP Biomedicals,LLC, Santa Ana, CA,USA). Twenty nanogram of each GST-fusion protein was incubated with 40,000–50,000 cpm of labeled probe in binding buffer (10% glycerol; 20 mM HEPES, pH 7.8; 45 mM KCl; 10 mM NaCl; 1 mM EDTA, pH 8.0; 1 mM DTT; 0.2 µg/µL BSA; and 0.1% nonidet P-40) with poly-dIdC (50 ng, Sigma-Aldrich Co., LLC, St. Louis, MO, USA) at room temperature for 30 min and then separated in a 6% polyacrylamide gel with 0.25×TBE (25 min prerun and then run at 4°Cat 120 V for 2.5 h). For heterodimerization, two proteins (each 20 ng) were preincubated in binding buffer at 4°C for 100 min before the addition of the probe. The reaction was extended for another h at 4°C and separated by electrophoresis. Radioactivity of the band was quantified with BAS4000 (FujiFilm Corporation, Tokyo, Japan).

### Immunohistochemistry

E18 mouse cortex was fixed with 4% paraformaldehyde overnight at 4°C and embedded in paraffin. The paraffin blocks were cut into 5 µm sagittal sections. After rehydration, the sections were stained with anti-PQBP1 that was produced in rabbit (1∶200, R00470, Sigma-Aldrich Co., LLC) and anti-Sox2 that was produced in goat (1∶200, Sox2-Y17, Santa Cruz Biotechnology, Inc.). The stains were visualized with AlexaFluor 488 in rabbit and Cy3 goat-conjugated secondary antibodies (1∶1,000, Dojindo Laboratories, Kumamoto, Japan). DAPI solution (1∶10,000, Dojindo Laboratories) was added to the secondary antibody solutionsin order tovisualize the nuclei. Digital images were captured by an Olympus IX71 microscope (Olympus Corporation, Tokyo, Japan) with a 10X/0.30objective lens (Figure 2B, 7) or a confocal microscopy (LSM510META, Carl Zeiss AG, Oberkochen, Germany) with 10X, 20X or 40X water emersion lens (Figure 1, 2A, 6B and supplementary figures). Image acquisition and immunofluorescent intensity quantification were performed with MetaMorph Image Browser software (Molecular Devices, LLC, Sunnyvale, CA, USA).

### Western Blot

Neurospheres prepared from E14 mouse telencephalon were dissolved in SDS sample buffer (62.5 mM Tris-HCl, pH 6.8; 2% SDS; 2.5% 2-mercaptoethanol; and 5% glycerol). Protein concentration was quantified with the BCA method (Micro BCA Protein Assay Reagent kit; Thermo Fisher Scientific Inc., Waltham, MA, USA). Twenty-five µg of proteins were applied for each lane. Anti-Sox2 produced in rabbit (ab5603, EMD Millipore Corporation) and anti-PQBP1produced in rabbit (R00470, Sigma-Aldrich Co., LLC) primary antibodies were used at 1∶500, and anti-GAPDH produced in mouse (MAB374, EMD Millipore Corporation) was used at 1∶10,000. Horseradish peroxidase-conjugated anti-Rabbit IgG (GE Healthcare) and horseradish peroxidase-conjugated anti-Mouse IgG (GE Healthcare) were used at 1∶3,000. Antibodies were diluted in TBS with Tween 20 (TBST) in 5% skim milk. ECL prime (GE Healthcare) was used to detect the bands according to the commercial protocol.

### Luciferase Assay

pGreenFire1-PC(Sox2+Brn2)-mCMV, pGreenFire1-NC(Sox2+Brn2)-mCMV, pGreenFire1-PC(Sox2+Pax6)-mCMV, or pGreenFire1-NC(Sox2+Pax6)-mCMV possessing active [29] or inactive cis-elements [43] were generated by annealing the tetramerized oligonucleotides into pGreenFire-1-mCMV reporter vector (System Biosciences, Mountain View, CA,USA). Similarly oligonucleotides of finally selected 18 *cis*-elements were subcloned into pGreenFire-1-mCMV reporter vector. The pGreenFire-1-mCMV reporter vector (200 ng), the pCI-neo effecter (pCI-neo-Sox2, Pax6 or Brn2∶20 ng), and pGL4.73 [hRluc/SV40] (Promega Corporation) (5 ng), which was used as an internal control, were cotransfected into P19 cells (4×10^4^/96-well plate) by lipofectamine 2000 (Life Technologies Corporation, Grand Island, NY, USA) according to the commercial protocol, when the cells became 80% confluent. Transfection was done at least in triplicate. Luciferase values were tested with a dual-glo luciferase assay system (Promega Corporation), and the average luciferase expression is presented with standard errors.

### In Utero Electroporation

For *in utero* electroporation, plasmids were prepared withan Endo-Free plasmid purification kit (QIAGEN Inc., Valencia, CA, USA). One µg of tetramerized pGreenFire1-PC (Sox2+Pax6)-mCMV or pGreenFire1-NC (Sox2+pax6)-mCMV [43] and pGreenFire1-16A-mCMVtogether with a DsRed expressing vector pLVSIN-CAG-pur-DsRed were electroporated into embryonic cerebral cortex at E14. The embryonic brain was dissected 24 h later and then fixed with 4% paraformaldehyde. Coronal tissue sections (200 µm) were visualized by confocal microscopy (LSM510META, CarlZeiss AG, Oberkochen, Germany).

### Ethics Statement

This study was performed in strict accordance with the recommendations in the Guide for the Care and Use of Laboratory Animals of the National Institutes of Health. The protocol was approved by the Committee on the Ethics of Animal Experiments of the Tokyo Medical and Dental University (Permit Number: 0130225A). All surgery was performed under ether of isoflurane anesthesia, and every effort was made to minimize suffering.

## Supporting Information

Figure S1 **Confirmation of the gel mobility shift assay conditions.** Positive control probes (Figure 4) bound to Sox2, Brn2, or Pax6 but not to GST protein.
(TIFF)

Figure S2 **Actual data of gel mobility shift assay in screening of cis-elements.** (A) Screening of *cis*-elements by a gel mobility shift assay with Sox2-Brn2 or Sox2-Pax6 full-length protein heterodimer. (B) Screening of cis-elements by a gel mobility shift assay with the Brn2 or Pax6 DNA-binding domain (DBD).
(TIFF)

Figure S3 **Screening of** ***cis*** **-elements by gel mobility shift assay with the Oct6 DNA-binding domain (DBD).** The left panel shows a representative gel mobility shift of the Sox2-Oct6 consensus probe by Oct6-DBD. A NF-κB consensus probe was used as a negative control. The graphs on the right show the radioactivity in the expected area of the gel shift of Oct6-DBD (surrounded by red line).
(TIFF)

Figure S4 **Locations of the double-positive sequences both upstream and downstream of the** ***PQBP1*** **gene.** The double-screening positive *cis*-elements were distributed both upstream and downstream of the *PQBP1* gene as clusters.
(TIFF)

Figure S5 **Larger magnification of** ***in utero*** **electroporated NSPCs.** (A) NSPCs transfected by pGreenFire1-16A-mCMV and pLVSIN-CAG-pur-DsRed were visualized by confocal microscopy with 40X water emersion lens. (B) A negative control plasmid, pGreenFire1-NC (Sox2+Pax6)-mCMV was also transfected into E14 embryonic brains by *in utero* electroporation. No EGFP signal was detected on E15.
(TIFF)
