# Supplementary figures and images for: Sox2 Transcriptionally Regulates Pqbp1, an Intellectual Disability-Microcephaly Causative Gene, in Neural Stem Progenitor Cells
Source: PLoS One. 2013 Jul 16;8(7):e68627. doi: 10.1371/journal.pone.0068627 (PMC3713010; doi:10.1371/journal.pone.0068627)

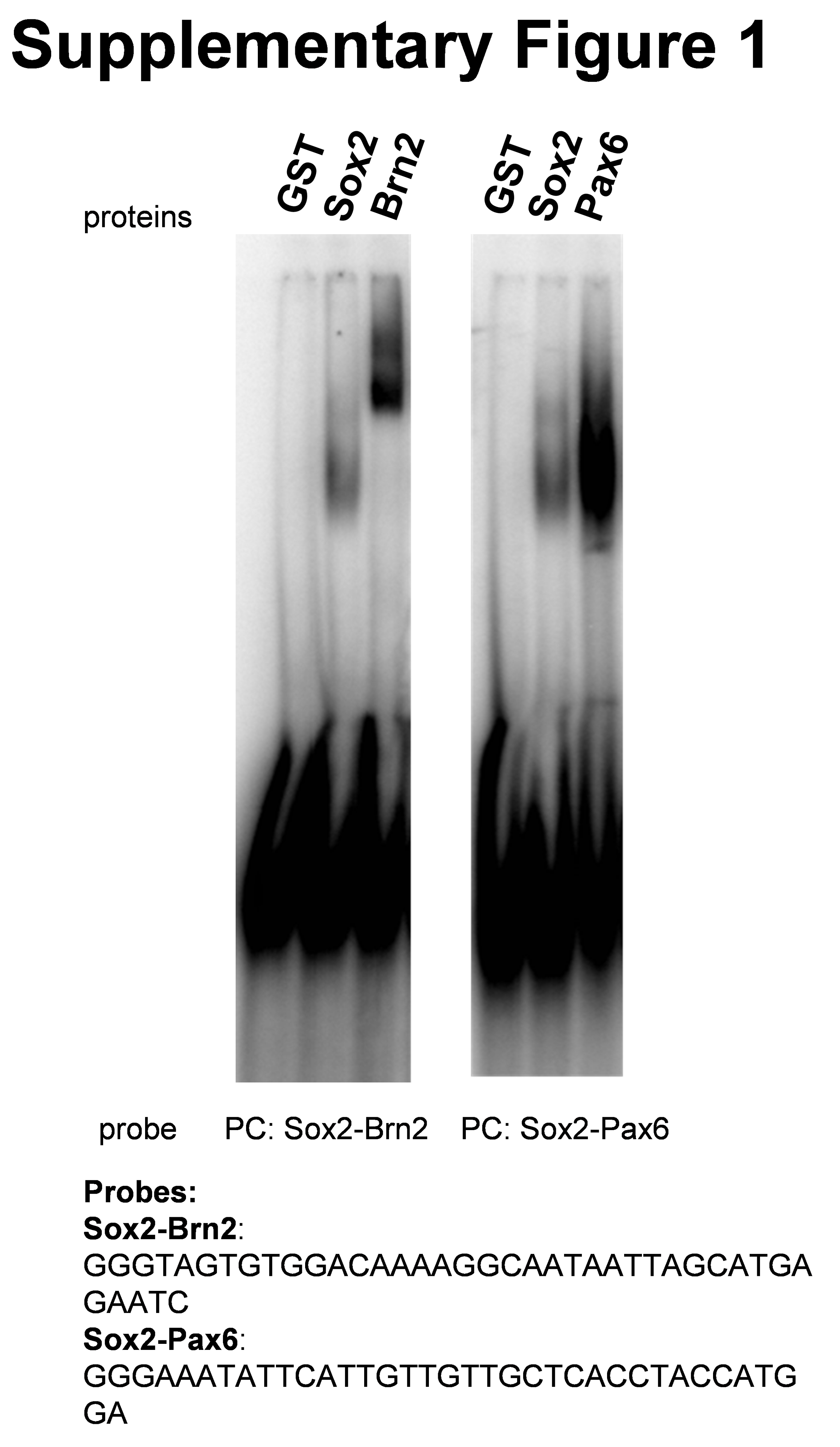

Supplement: Figure S1 — Confirmation of the gel mobility shift assay conditions. Positive control probes (Figure 4) bound to Sox2, Brn2, or Pax6 but not to GST protein. (TIFF) [file pone.0068627.s001.tiff]

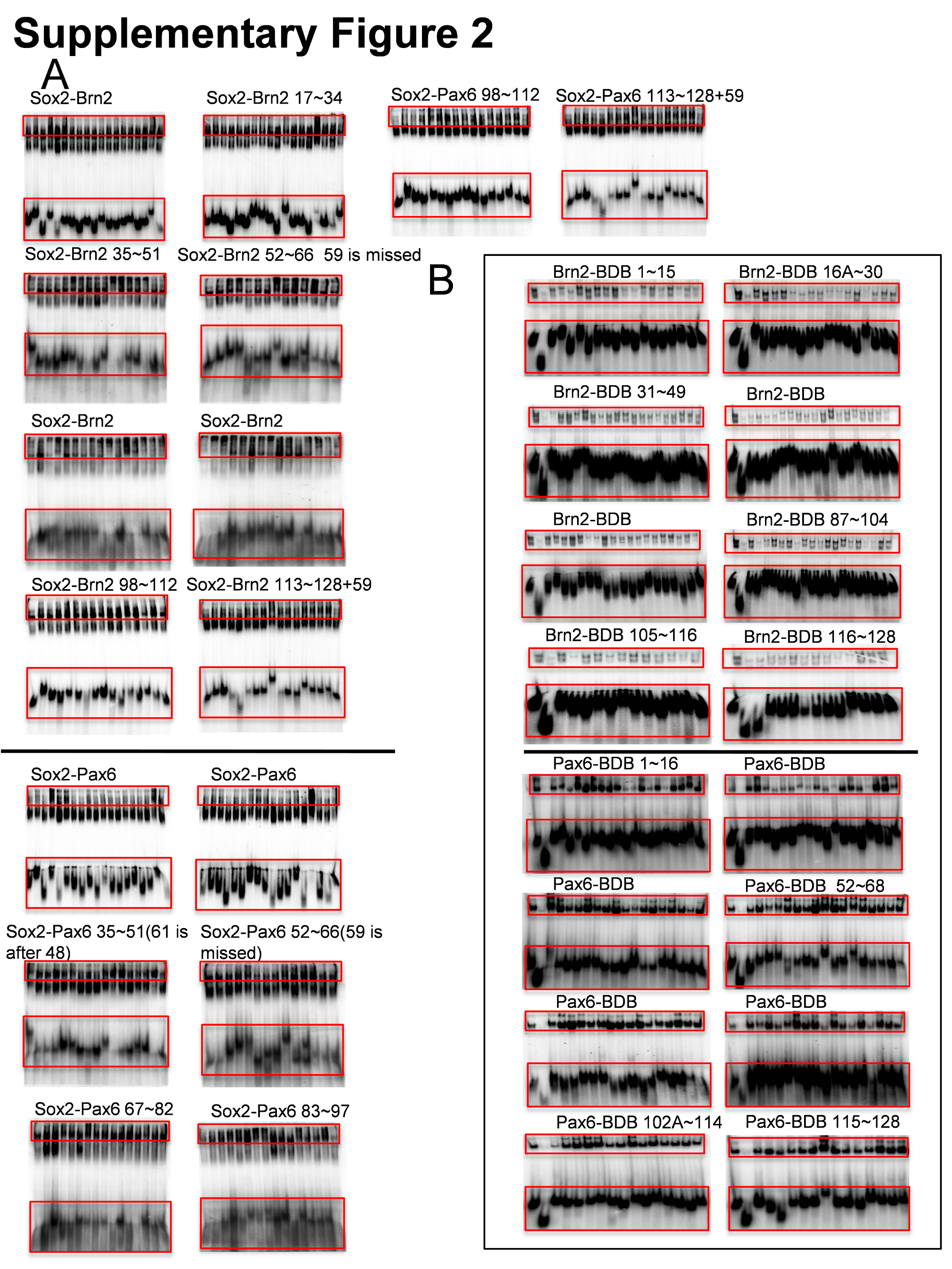

Supplement: Figure S2 — Actual data of gel mobility shift assay in screening of cis-elements. (A) Screening of cis-elements by a gel mobility shift assay with Sox2-Brn2 or Sox2-Pax6 full-length protein heterodimer. (B) Screening of cis-elements by a gel mobility shift assay with the Brn2 or Pax6 DNA-binding domain (DBD). (TIFF) [file pone.0068627.s002.tiff]

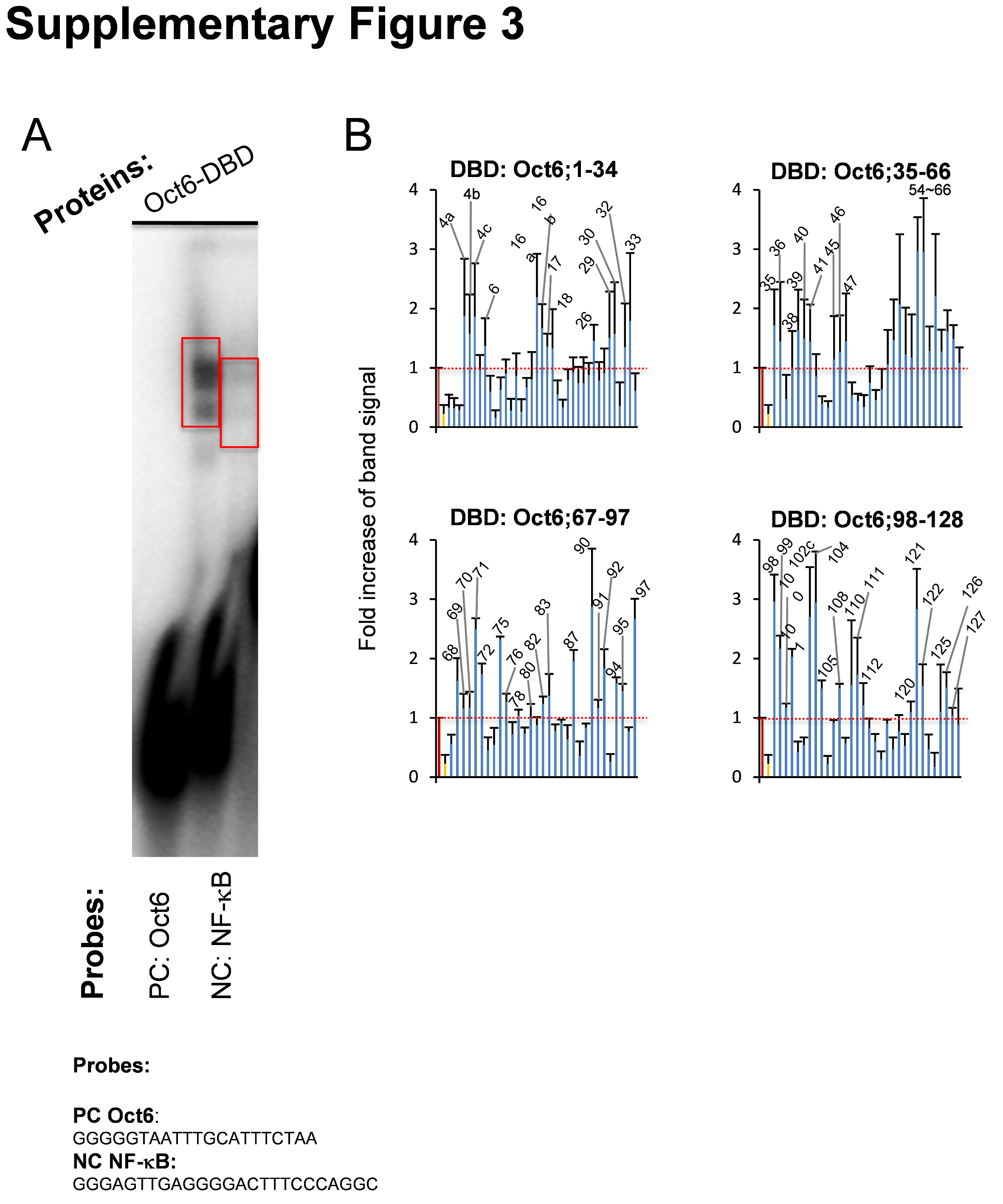

Supplement: Figure S3 — Screening of cis -elements by gel mobility shift assay with the Oct6 DNA-binding domain (DBD). The left panel shows a representative gel mobility shift of the Sox2-Oct6 consensus probe by Oct6-DBD. A NF-κB consensus probe was used as a negative control. The graphs on the right show the radioactivity in the expected area of the gel shift of Oct6-DBD (surrounded by red line). (TIFF) [file pone.0068627.s003.tiff]

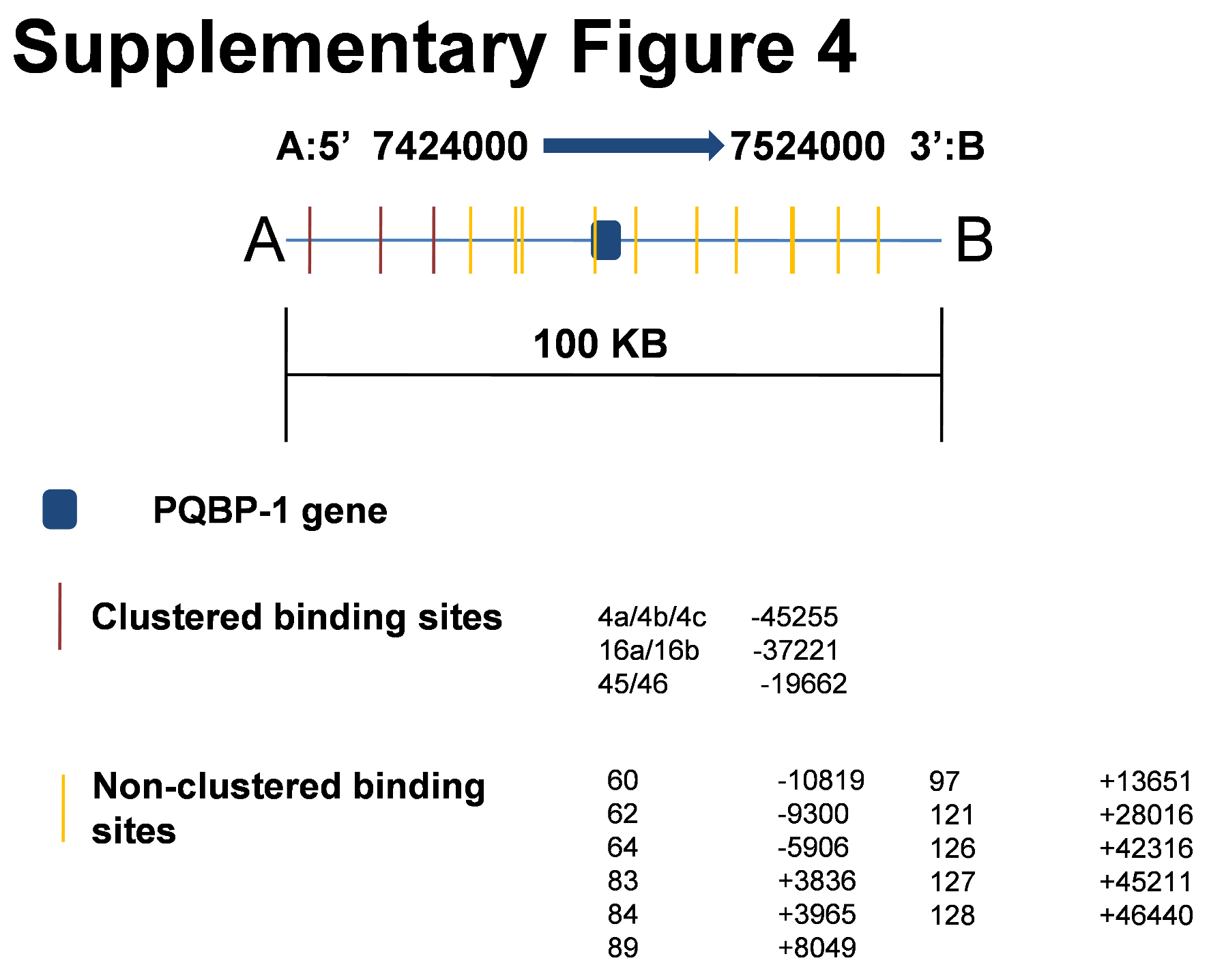

Supplement: Figure S4 — Locations of the double-positive sequences both upstream and downstream of the PQBP1 gene. The double-screening positive cis-elements were distributed both upstream and downstream of the PQBP1 gene as clusters. (TIFF) [file pone.0068627.s004.tiff]

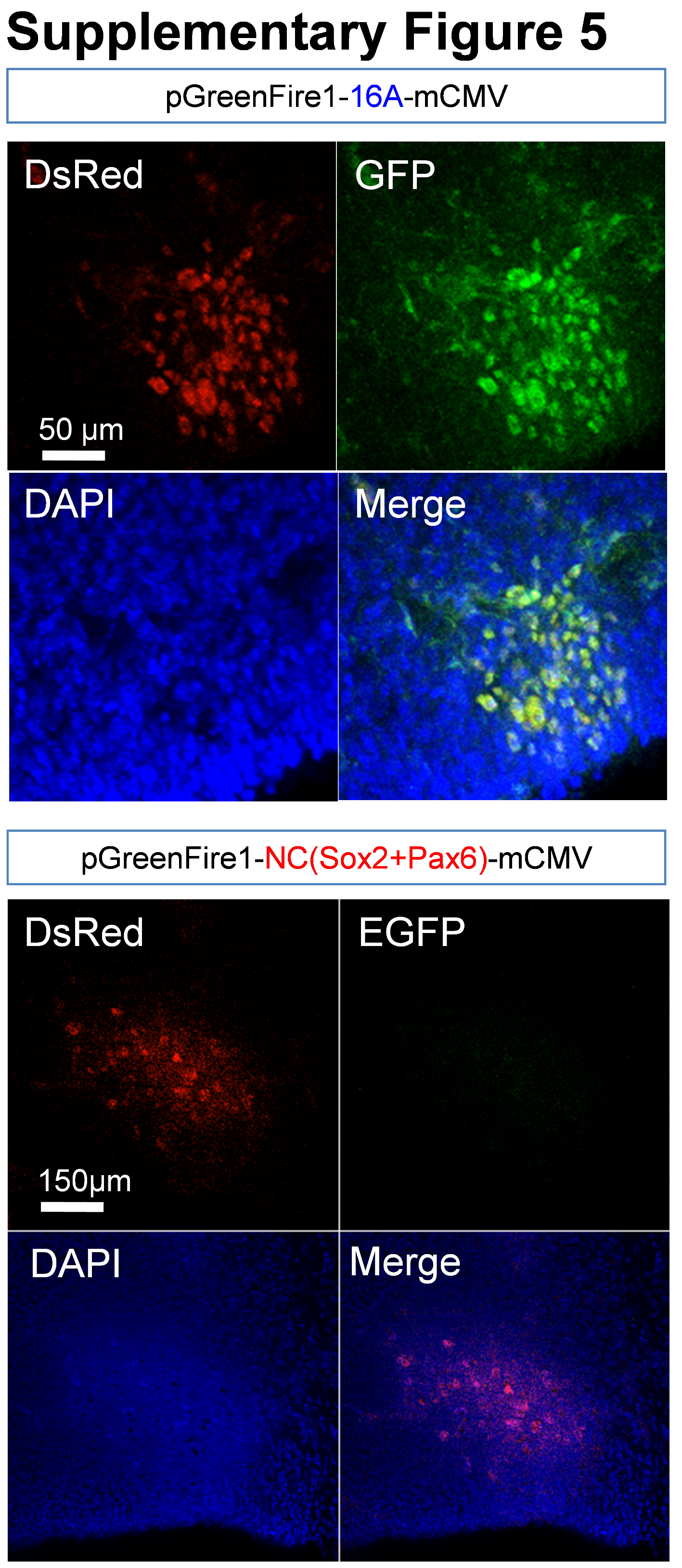

Supplement: Figure S5 — Larger magnification of in utero electroporated NSPCs. (A) NSPCs transfected by pGreenFire1-16A-mCMV and pLVSIN-CAG-pur-DsRed were visualized by confocal microscopy with 40X water emersion lens. (B) A negative control plasmid, pGreenFire1-NC (Sox2+Pax6)-mCMV was also transfected into E14 embryonic brains by in utero electroporation. No EGFP signal was detected on E15. (TIFF) [file pone.0068627.s005.tiff]
